# A secure and efficient certificateless content extraction signature with privacy protection

**DOI:** 10.1371/journal.pone.0258907

**Published:** 2021-11-01

**Authors:** Can Zhao, Jiabing Liu, Fuyong Zheng, Dejun Wang, Bo Meng

**Affiliations:** 1 School of Computer Science, South-Central University for Nationalities, Wuhan, China; 2 Information and communication Branch, Jiangxi electric power Co. ltd., Nanchang, China; University College of Engineering Tindivanam, INDIA

## Abstract

Efficiency and privacy are the key aspects in content extraction signatures. In this study, we proposed a Secure and Efficient and Certificateless Content Extraction Signature with Privacy Protection (SECCESPP) in which scalar multiplication of elliptic curves is used to replace inefficient bilinear pairing of certificateless public key cryptosystem, and the signcryption idea is borrowed to implement privacy protection for signed messages. The correctness of the SECCESPP scheme is demonstrated by the consistency of the message and the accuracy of the equation. The security and privacy of the SECCESPP scheme are demonstrated based on the elliptic curve discrete logarithm problem in the random oracle model and are formally analyzed with the formal analysis tool ProVerif, respectively. Theory and experimental analysis show that the SECCESPP scheme is more efficient than other schemes.

## Introduction

Content extraction signatures have been widely used to protect electronic medical records and electronic commerce security, in which the signature verifier can verify the authenticity of the extracted message without knowing the entire signed message [1]. Efficiency and privacy protection are the key aspects of a content extraction signature. For efficiency purposes, most content extraction signature schemes were developed using the traditional public key cryptosystem [2–5] or the identity-based public key cryptosystem [6–10]. Owning to the problems of certificate management in traditional public key cryptosystems and user key management in identity-based public key cryptosystems, respectively, people have turned to certificateless public key cryptosystems to implement the content extraction signatures [11–13] in the present day because no certificate is required and key management problems barely exist for certificateless public key cryptosystem. However, certificateless public key cryptosystem are based on costly bilinear paring of elliptic curves. The efficiency of such an approach is low. The operations currently available for elliptic curves include exponential operation, scalar multiplication, bilinear pairing and a hash function According to references [8,9], scalar multiplication is the most efficient operation and is therefore a good option to replace inefficient bilinear pairing in certificateless public key cryptosystem.

For privacy protection, the signed message of a content extraction signature may contain a private message. However, existing content extraction signatures do not consider privacy protection. The available privacy protection methods mainly include data distortion and data encryption [11]. Data distortion has no universality and depends on the data types used. Data encryption is a good choice for implementing privacy with regard to content extraction signatures. In general, the sign-then-encrypt approach can provide privacy and authentication. However, it is inefficient because the encryption and signature processes are separate operations. But signcryption [2] can provide privacy and authentication with high efficiency because the encryption and signature are completed in a single logical step.

Therefore, to improve the efficiency of and provide privacy protection for content extraction signatures, we propose a Secure and Efficient Certificateless Content Extraction Signature with Privacy Protection (SECCESPP) scheme. The main works is presented as follows:

1. Scalar multiplication is used on elliptic curves to replace inefficient bilinear pairing in a certificateless public key cryptosystem, and the signcryption idea from data encryption approach is borrowed to implement privacy for signed message.
2. The correctness of the scheme is demonstrated by the consistency of the message and the accuracy of the equation, the scheme’s security is verified based on the elliptic curve discrete logarithm problem in the random oracle model, and privacy is analysed with the formal analysis tool ProVerif.
3. The scheme is compared to the related schemes [14–19], revealing that the SECCESPP scheme is more efficient.

The rest of the paper is organized as follows. Section 1 discussed the related works of privacy protection, content extraction signature and signcryption. Section 2 makes a simple review of preliminaries. Section 3 presents the SECCESPP scheme. Section 4 illustrates the correctness of the SECCESPP scheme. Section 5 gives proofs about security requirements. Section6 makes comparisons of performance. Finally, Section 7 presents the conclusion and future work.

## Related work

Since the SECCESPP scheme involves privacy protection, content extraction signatures and signcryption, this section briefly introduces the related works regarding these three components.

### Privacy protection

There is much information that is vulnerable to attackers. In 2017, Adat et al. discussed the history, background, statistics of IoT and security based analysis of IoT architecture and provided taxonomy of various defense mechanisms [20]. It is urgent to protect private information. The existing privacy protection technologies can be divided into two categories: data distortion and data encryption approaches [11].

Technologies based on data distortion distort sensitive data while keeping some data or data attributes unchanged, and the most common technique of this type is the differential privacy technique. In 2018, Ye [21] proposed a new localized differential privacy protection model. The protection model adopts random response technology, which first makes the data private and then sends it, to provide comprehensive protection for sensitive information; this approach can not only resist attackers with arbitrary background knowledge but also prevent privacy attacks from distrusted third parties. However, it depends heavily on the accompanying data, and different algorithms are designed for different data, so it has no universality. In 2021, Stergoiu et al. [22] proposed an innovative system of secure caching scenario which operates in a wireless-mobile 6G network for managing BD on Smart Buildings(SB) and created a novel and secure Cache Decision System(CDS) in a wireless network that operates over a SB, which offer the users a safer and efficient environment for browsing the internet, sharing and managing large-scale data in the fog. It could be a start point for better and more efficient wireless networking scenario, for managing and sharing Big Data on a Smart Building.

Technologies based on data encryption hide sensitive data in the process of data mining. It is mainly used in distributed application environments and can solve the communication security problem. At present, Homomorphic Encryption (HE) is widely used. HE refers to specific classes of encryption schemes that allow for computing directly on encrypted data without having to decrypt them. In 2020, Zhao [23] presented a circular secure public key homomorphic encryption scheme using noise flooding technique, and provided security proofs and parameter setting. Furthermore, by introducing the refuse sampling technique, an optimized circular secure public key homomorphic encryption scheme was given, and the system parameters were reduced from the super-polynomial level to the polynomial level, thereby greatly reducing the size of the public key and ciphertext. Then, the computational complexity of ciphertext evaluation could be effectively improved, and the performance of the homomorphic encryption scheme could be enhanced. However, at the same time, there are some problems with this method, such as high computing costs, high communication costs, complex deployment, and high practical application difficulty. In 2021, Zhang et al. [24] proposed a secure decentralized spatial crowdsourcing scheme for 6G-Enabled Network in Box using CBC-MAC authenticated encryption mechanism to provide confidentiality and integrity. It solves leakage of sensing nodes locations. But it Still hasn’t solved the problem of data leaks in transit. In the same year, in order to solve the security of shared information VANET system, Vijayakumar et al. [25] proposed an efficient batch authentication and key exchange schemes, which will be applied to blockchain users in the future. Then, Azees et al. [26] completed the following work, applying blockchain technology to the security guarantee of VANET system, realizing the rapid reauthentication of vehicles, and making a contribution to the information security in the future blockchain era.

### Content extraction signature

The Content Extraction Signature (CES) was first proposed by Steinfeld [27]. According to the technology used, CES types are mainly divided into a CommitVector (CV)-based content extraction signature, an RSA-based content extraction signature, and a hash tree (HT)-based content extraction signature.

A CES scheme [3] based on a CV has the characteristics of unforgeability and exclusivity. Its unforgeability is jointly guaranteed by the standard digital signature EUF-CMA and the binding of the message commitment scheme, while its exclusivity is guaranteed by the hiding of the message commitment scheme. Scheme [27] formalized and proved these two securities. Scheme [3] has a lower computational cost than the CES scheme [27], which requires a signature operation and a commitment operation to be performed for the original signature generation process. However, because the original signature and the intercepted signature contain the committed random numbers of all the retained sub-data and the committed values of the deleted sub-data, the length of the signature expands, and the communication overhead increases.

To solve the problem of CVs, an RSA-based CES was proposed. An RSA-based CES is formed on the basis of a CV-based CES using the RSA signature, and the length of the signature is only the length of the modulo of the RSA signature, which greatly reduces the length of the signature. Combining this with the idea of batch signatures, in 2014, Li [4] proposed an improved scheme for content extraction signatures based on RSA. The scheme can judge whether a content extraction access structure (CEAS) meets the given extraction conditions through the correspondence between (*M*′) and *CI*(*M*′). In 2015, Lan [28] proposed an identity-based CES scheme. This scheme does not need to sign every sub-message, thereby improving efficiency, and it can prevent PKG from forging signatures and thus improve its application value. In 2017, Wang [29], based on [28], achieved the goal of shortening the length of the signature by reducing the commitment value and random number and performed unified signature and verification operations on sub-messages, which improved the efficiency of signing and verification.

However, by using quantum cryptography, the keys of Std.RSA might get broken down to approximately 850 bits. This result in the need to enhance the current public key cryptosystem. Thus, the HT-based CES was proposed. Drawing on the idea of a binary tree, hashing every two message blocks generates a commitment hash value, recursively, layer by layer, and obtains a total hash value, which greatly reduces the chance of the CES breaking. In 2016, Thirumalai [30] proposed a commitment tree-based batch signature scheme. Compared with the CV-based CES and RSA-based CES, this scheme has a lower signature length, fewer calculation operations, and improved signature efficiency. In 2019, Szalachowski [31] proposed a TLS-N method based on the TLS extension. In this method, the Merkle tree is used during the process of generating evidence. The server generates evidence about the TLS session content, generates a noninteractive certificate about the TLS session content on the client, and then sends the session content and the certificate to a third party for verification. In 2020, Cheng [32] proposed a blockchain based secure storage and sharing scheme for electronic health records data. In this scheme, a certificateless content extraction signature algorithm is used to provide privacy protection, secure sharing of data has realized in combination with smart contracts. The combination of blockchain and content extraction signature is better applied in the electronic medical records.

### Signcryption

Signcryption is a cryptographic primitive that captures a common practical scenario where one simultaneously requires confidentiality and nonrepudiation for transmitted data. Signcryption schemes achieve confidentiality and authentication simultaneously by combining public key encryption and digital signatures, offering better overall performance and security than other schemes [11]. There are three types of signcryption: public key infrastructure (PKI)-based signcryption, identity (ID)-based signcryption, and certificateless signcryption.

A PKI is required to manage and distribute public keys. In such systems, a public key is bound to the corresponding unique user *ID*. Trusted third-party tools are used to bind the users to unique public keys through an appropriate registration process. Based on the PKI concept, in 1997, Zheng [2] proposed signcryption, which has since been widely discussed and studied. The original scheme used interactive zero-knowledge proof technology, which is not efficient. In 2019, Yan [5] proposed a signcryption scheme that directly uses the sender’s public key to verify the validity of the signature. Compared with the sign-then-encrypt mechanism, the public key size and the computational cost of the signcryption operation are both obviously reduced. However, the use of an additional third-party application makes public key cryptography expensive and inefficient.

To overcome the problem of the PKI management system, many ID-based signcryption schemes have been proposed. The idea of ID-based signcryption was first proposed by Malone [6] in 2002 along with a security model. This model was developed by Boyen [7]. Three new security notions were added: ciphertext unlinkability, ciphertext authentication and ciphertext anonymity. In 2019, Wang [8] proposed a basic model for ID-based signcryption schemes that can use bilinear pairs to design signcryption schemes. In the same year, Shankar [8] pointed out that scheme [8] was not secure and proposed three new secure solutions. However, they do not satisfy public verifiability and forward security at the same time. In response to this problem, in 2019, Pan [10] proposed a solution that uses two private keys for signcryption and unsigncryption. In 2019, Deng [33] proposed a new ID-based signcryption model, that solved the problem in which [9] does not simultaneously satisfy public verifiability and forward security. However, the system needs a third-party application for private key management to generate them secretly and distribute them to users.

To address PKI-based and ID-based signcryption issues, a certificateless signcryption approach was proposed by Riyamin [11] in 2008. It presents stronger security properties than one might expect from its internal building blocks; sharing randomness between encryption and signature modules not only provides extra savings in terms of the computational and bandwidth loads but also yields strong insider security guarantees. In 2019, Gao [12] proposed an improved certificateless signcryption scheme. This scheme guarantees the security of the signcryption phase by defining the length of the message space during the system establishment phase. In the same year, Wang [13] proposed the definition of a blind signcryption scheme under certificated and certificateless public key cryptosystems and proposed a blind signcryption scheme based on bilinear pairing. The scheme increases blindness, but the computational cost does not increase significantly. In 2020, Fang [34] proposed a certificateless multi-receiver multi-message simultaneous broadcast signcryption scheme. Combined with random elements in an elliptic curve cyclic group, the encryption key is generated, which solves the problems of receiver decryption ciphertext and identity anonymity protection. However, the scheme lacks a security mechanism when verifying the signcryption.

## Preliminaries

This section introduces the commitment scheme, salt tree, binary commitment tree and content extraction access structure used in the SECCESPP scheme.

### Commitment

Commitment, with two characteristics of Hiding and Binding, is a fundamental model in the field of cryptography. Hiding means that the commitment can hide information, that is, no other entity can obtain information from the commitment except the entity that places the information there. Binding means that no entity is allowed to change the information within the commitment, and it can verify that the information it receives is indeed the information originally promised.

The commitment scheme is composed of three algorithms: *Gen*(), *Com*() nad *Ver*().

Initialization phase: *Gen*() accepts a “1” bit string of length *k* as input and outputs a common reference string *crs*.


$$\text{c}rs\leftarrow Gen(1^{k})$$ (1)

Commitment phase: *Com*() accepts a common reference string *crs* and a committed message *m* as input, and outputs *m*’s commitment value *com* and decommitment information *dec*.


(com,dec)←Com(crs,m) (2)

Decommitment phase: The sender sends *dec* and *m* to the receiver.

Verification phase: *Ver*() accepts the message *m*, common string *crs*, commitment value *com* and decommitment information *dec* as input, and it outputs verification result.


Yes/No←Ver(crs,com,dec,m) (3)

In the SECCESPP scheme, the entire message *M* is divided into n blocks, *M* = {*M*[1], *M*[2], … *M*[*i*], …, *M*[*n*]}, along with a random number string called salt, and used as input for the message commitment algorithm *C*(). Because the entropy of the message block is low, pseudorandom salt can be used to protect the committed privacy and avoid brute force attacks. Combine the message block *M*[*i*] and pseudorandom salt $S_{i_{r},i_{c}}$ together to generate the commitment $c=C(M[i],S_{i_{r},i_{c}})$. Recalculate the commitment $c\prime =C(M\prime [i],S_{i_{r},i_{c}})$ for the new message *M*′[*i*] during the verification phase. If *c* = *c*′ is true, the new message *M*′[*i*] is indeed the message *M*[*i*] originally promised.

### Salt tree

The leaf nodes in a salt tree are used as salt to generate a binary commitment tree to greatly increase the computations required by an attack.

To protect privacy, $S_{i_{r},i_{c}}$ and $S_{i_{r}^{\prime },i_{c}^{\prime }}$ must remain independent, meaning that the salt tree generation process cannot be sent directly to the verifier and $S_{i_{r},i_{c}}$ must be retrieve. The salt tree is constructed by the function *E*(), and the salt value is obtained as the input of the binary commitment tree. The inputs for the function *E*() are a session-based secret value and a Nonce random value, and the output is a pseudorandom salt. During the verification process, the corresponding salt is required for completion.

### Binary commitment tree

The purpose of building the binary commitment tree is to include the commitment in the proof. The commitment value generated by the commitment algorithm serves as input for obtaining the leaf node value of the binary commitment tree. The hash value *h*_*i*_ of the binary commitment tree is calculated by the anti-collision hash function of the hash value, session message length *l*_*r*_ and signer information of *O*_*i*_ its child node. *O*_*i*_ is the i-th member of the order vector. The commitment values generated in section II.B are used as leaf nodes to generate the hash chain. All the leaf nodes can be verified by signature verification of the root node using the binary commitment tree.

The generation process for a binary commitment tree is as follow: A session message Record *i* consists of *n* sub-message blocks. Each message block and its corresponding salt leaf node *salt* secret serve as input; the commitment value *c* is generated as the leaf node of the binary commitment tree. Every two leaf nodes are hashed in a cascade recursively, layer by layer, until the root node is finally obtained. At this point, the binary commitment tree construction process is completed.

### Content extraction access structure

To extract sub-message blocks from the original message, the concept of a content extraction access structure (CEAS) is introduced. Sub-message block numbers in a CEAS must be extracted after the original message is partitioned. For example, {*M*[1], *M*[2], *M*[3], *M*[4]} means that the original message *M* is divided into four sub-message blocks; *CEAS* = {1,3} means that sub-message blocks *M*[1] and *M*[3] must be extracted, and sub-message blocks *M*[2] and *M*[4] can be extracted. If *CI*(*M*′) = {1, 3, 4}, then *M* = {*M*[1],?, *M*[3], *M*[4]}, where ‘?’ represents the unextracted sub-message block, which is denoted as *CEAS* ∈ *CI*(*M*′). Such an extraction method is legal. If *CI*(*M*′) = {3, 4}, then M′ = {?,?, *M*[3], *M*[4]}, and this is represented as *CEAS* ∉ *CI*(*M*′); this kind of extraction method is illegal.

## The SECCESPP scheme

The SECCESPP scheme can implement a content extraction signature with privacy protection for a singed message in a single security mechanism in which scalar multiplication on elliptic curves and the signcryption idea are used.

**Fig 1:**
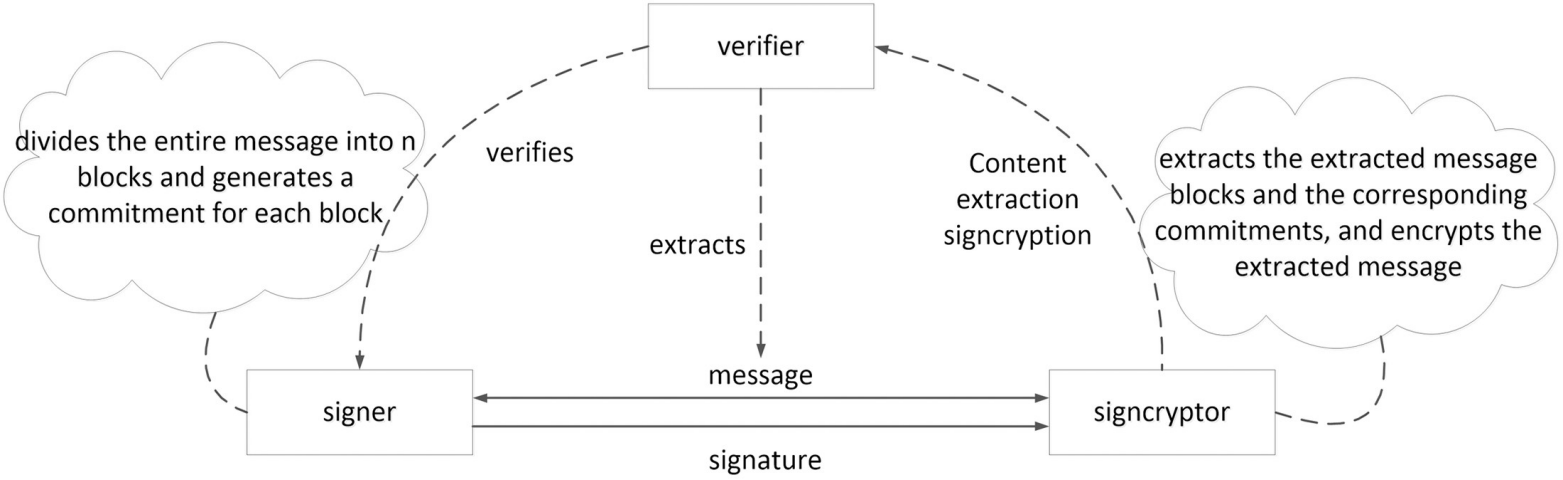
The main research framework of the SECCESPP scheme.

Fig 1 shows the main research framework of the SECCESPP scheme. The SECCESPP scheme is composed of Key-Generation, Signature-Generation, Signcryption-Extraction, and Signcryption-Verification algorithms. Three roles are defined: signer, signcryptor and verifier. The signer first divides the entire message into *n* blocks and then generates a commitment for each block. Next, the signer generates a signature and sends it to the signcryptor. After receiving the signature, the signcryptor extracts the extracted message blocks and the corresponding commitments, encrypts the extracted message, and generates the content extraction signcryption sent to the verifier. The verifier receives and verifies the content extraction signcryption.

### Key-Generation algorithm

The Key-Generation algorithm generates a public key *SK*_*A*_ and a private key *PK*_*A*_ for signer A in a certificateless cryptosystem.

1. Set up the system parameters: First, the KGC (key generator center) selects the master number of k-bits *P*, where *k* is the security parameter, and obtains {*F*_*P*_, *E* / *F*_*P*_, *G*, *P*}. Then, $x\in Z_{n}^{*}$ is selected as the system master key *msk*, and the master public key *P*_*pub*_ = *xP* is calculated. Next, hash function are selected:$H_{1}:{\left\{0,1\right\}}^{*}\to Z_{n}^{*}$, *H*_2_: {0, 1}* → {0,1}^*k*^, *H*_3_: {0, 1}* → {0,1}^*k*^ and $H_{4}:{\left\{0,1\right\}}^{*}\to Z_{n}^{*}$. The KGC saves *params* = {*F*_*p*_, *E* / *F*_*p*_, *G*, *P*, *H*_1_, *H*_2_, *H*_3_, *H*_4_}.
2. Signer A randomly selects $x_{A}\in Z_{n}^{*}$ as the secret value, computes *P*_*A*_ = *x*_*A*_ · *P*, and sends it to the KGC, where the identity of signer A is *ID*_*A*_. The KGC calculates *h*_*A*_ = *H*_1_(*ID*_*A*_, *R*_*A*_, *P*_*A*_), *R*_*A*_ = *r*_*A*_ · *P* and *s*_*A*_ = *r*_*A*_ + *h*_*Ax*_ mod *n* to generate a partial key *D*_*A*_ = {*S*_*A*_, *R*_*A*_}. Finally, the private key *SK*_*A*_ = (*x*_*A*_, *s*_*A*_) and public key *PK*_*A*_ = (*P*_*A*_, *R*_*A*_) are produced for signer A in a certificateless public cryptosystem.

### Signature-Generation algorithm

The Signature-Generation algorithm generates the signature *σ*_*F*_ for the entire message *M*. The entire message *M* is divided into n blocks, *M* = {*M*[1], *M*[2], … *M*[*i*], …, *M*[*n*]}. *M*′ is any submessage extracted from *M*.*ext*(*i*) represents the number of submessage blocks that are common in *M* and *M*′. *CEAS* is a content extraction access structure. The complete pseudocode for signature generation is given in Algorithm 1.

**Algorithm 1** Signature-Generation

**Input**: *params*, *ID*_*A*_, *SK*_*A*_, *PK*_*A*_, *M*, *CEAS*

**Output**: signature *σ*_*F*_

1: **repeat**

2:  $c[i]=C(M[i],S_{i_{t}i_{c}})$, *a*[*i*] = *A*_*n*_[*i*]l *v*_*a*_[*i*] = *c*[*i*]

3: **until** *i* > *n*

4:  **while** *a* ∈ *I*_*n*_ **do** *v*_*a*_ = H_2_ (v_a,0_, v_a,1_)

5:  **end while**

6: **choose** $l\in Z_{n}^{*}$, *R* = *l* · *P*

7: *h* = *H*_3_ (*v*_0_, *R*, *PK*_*A*_)

8: **if** *gcd*(*l* + *h*, *n*) = 1 **then**

9:  *s* = (*l* + *h*)^−1^ (*x*_*A*_ + *s*_*A*_)mod *n*

10: **end if** *σ*_*F*_ = (*CEAS*, *R*, *s*, *c*[*i*]_i∈n_)

11: **return** *σ*_*F*_ //generates signature

### Signcryption-Extraction algorithm

After receiving the signature *σ*_*F*_, the signcryptor obtains *v*_0_ according to the Signature-Generation algorithm, calculates *h*_*A*_ = *H*_1_(ID_A_, *R*_*A*_, *P*_*A*_) and *h* = *H*_3_(*v*_0_, *R*, *PK*_*A*_), and verifies the equation *s*(*R*+ *hP*) = *P*_*A*_ + *R*_*A*_ + *h*_*A*_*P*_*pub*_. If the equation is not correct, the signcryptor stops the algorithm. Otherwise, the following steps are performed to obtain the signcryption *σ*_*E*_:

1. Generate *ext*(*i*) according to the *CEAS*.
2. Extract *M*′ = {*M*′[1], *M*′[2], *M*′[3], …, *M*′[*i*], …, *M*′[*n*]}. The specific measures are as follows: if *i* ∈ *ext*(*i*), *M*′[*i*] = *M*[*i*], indicating that the submessage block is extracted; Otherwise, *M*′[*i*] = *c*[*i*].
3. Calculate *E*_*A*_, *E* and extract the signcryption *σ*_*E*_

The complete pseudo code for signcryption extraction is given in Algorithm 2.

**Algorithm 2** Signcryption-Extraction

**Input**: message *M*, signature *σ*_*F*_, *ext*(*i*), *PK*_*A*_

**Output**: signature *σ*_*E*_

1: (*CEAS*, *R*, *s*, *c*[*i*]_*i*∈*n*_) ← *σ*_*F*_

2:  **repeat**

3:   *v*_*a*_[*i*] = *c*[*i*]

4:   **while** *a* ∈ *I*_*n*_ **do** *v*_*a*_ = H_2_(*V*_*a*,0_, *V*_*a*,1_)

5:   **end while**

6:  *h*_*A*_ = *H*_1_(*ID*_*A*_, *R*_*A*_, *P*_*A*_), *h* = *H*_3_(*v*_0_, *R*, *PK*_*A*_)

7:  **if** *s*(*R* + *hP*) = *P*_*A*_ + *R*_*A*_ + *h*_*A*_*P*_*pub*_ **then**

8:    **if** *i* ∈ *ext*(*i*) **then** //generates *ext*(*i*)

9:    *M*′[*i*] = *M*[*i*]

10:   **else** *M*′[*i*] = *c*[*i*]

11:   **end if**

12:  **end if**

13:  *E*_*A*_ = *l*(*P*_*A*_ + *R*_*A*_ + *h*_*A*_*P*_*pub*_), *E* = *H*_4_(*E*_*A*_) ⊕ *M*′

14:  *σ*_*E*_ = (*E*, *CEAS*, *ext*(*i*), *R*, *s*)

15:  **return** *σ*_*E*_

### Signcryption-Verification algorithm

After receiving the signcryption *σ*_*E*_, the verifier decrypts *M*′and *v*_0_ and verifies the signcryption by the equation *s*(*R* + *hP*) = *P*_*A*_ + *R*_*A*_ + *h*_*A*_*P*_*pub*_. The complete pseudocode for signcryption verification is given in Algorithm 3.

**Algorithm 3** Signcryption-Verification

**Input**: submessage *M*′, signcryption *σ*_*E*_, *PK*_*A*_

**Output**: verification result

1:  **if** *ext*(*i*) ∈ *CEAS* **then** //else, stops the algorithm

2:  *E*_*B*_ = *s*(*x*_*A*_ + *s*_*A*_)(*P*_*A*_ + *R*_*A*_ *R* + *h* · *p*)

3:   *M*′ = *E* ⊕ *H*_4_(*E*_*B*_) //decrypts *M*′

4:  **repeat**

5:   *c*[*i*] = *C*(*M*[*i*], *S*_*iR*,*ic*_), *a*[*i*] = *A*_*n*_[*i*]; *v*_*a*_[*i*] = *c*[*i*]

6:   **while** *a* ∈ *I*_*n*_ **do**

7:    *v*_*a*_ = *H*_2_(*v*_*a*,0_, *v*_*a*,1_) //gets (decrypts) *v*_0_

8:   **end while**

9:  *h*_*A*_ = *H*_1_(*ID*_*A*_, *R*_*A*_, *P*_*A*_), *h* = *H*_4_(*v*_0_, *R*, *PK*_*A*_)

10:  **if** *s*(*R* + *hP*) = *P*_*A*_ + *R*_*A*_ + *h*_*A*_*P*_*pub*_ **then**

11:   **return Acc** // *σ*_*E*_ verified successfully

12:  **else**

13:   **return Rej** // *σ*_*E*_ verification failed

## Correctness analysis

In this section, we prove the correctness by the consistency of the message and the accuracy of the equation.

### Message consistency

Message consistency indicates that the extracted message *M*′ in the Signcryption-Extraction algorithm is consistent with the decrypted message *M*′ in the Signcryption-Verification algorithm.

We analyze the consistency between the submessage *M*″ extracted in the Signcryption-Extraction algorithm and the submessage *M*‴ decrypted in the Signcryption-Verification algorithm.

Submessage *M*″ is extracted in the Signcryption-Extraction algorithm using the following equation:

$$M\prime \prime [i]=\{\begin{array}{cc} M[i], & i\in ext(i)\\ c[i]=C(M[i],S_{i_{r},i_{c}}), & i\notin ext(i) \end{array}$$ (4)

Submessage *M*″ is decrypted in the Signcryption-Verification algorithm using the following equation:

$$M\prime \prime \prime [i]=\{\begin{array}{cc} c[i]=C(M[i],S_{i_{r},i_{c}}), & i\notin ext(i)\\ M[i], & i\in ext(i) \end{array}$$ (5)

The fact is that *M*″ and *M*‴ are the same, hence, the SECCESPP scheme has consistency.

### Equation accuracy

If equation *s*(*R* + *hP*) = *P*_*A*_ + *R*_*A*_ + *h*_*A*_*P*_*pub*_ in the Signcryption-Verification algorithm is true, then the SECCESPP scheme is bounded. In this section, we check *s*(*R* + *hP*) = *P*_*A*_ + *R*_*A*_ + *h*_*A*_*P*_*pub*_ with the following process.


$$\begin{array}{l} ∵Ppub=x\cdot P,s_{A}=r+h_{A}x\;\text{mod}\;n\\ ∴s_{A}\cdot P=R_{A}+h_{A}\cdot P_{pub}\\ ∵s={\left(l+h\right)}^{-1}(x_{ID}+s_{ID})\;\text{mod}\;n\\ \begin{array}{cc} ∴left & =s(R+hP)={\left(h+l\right)}^{-1}(x_{A}+s_{A})(l+h)P\\ & =x_{A}\cdot P+s_{A}\cdot P=P_{A}+R_{A}+h_{A}\cdot P_{pub}=right \end{array} \end{array}$$ (6)

Therefore, *s*(*R* + *hP*) = *P*_*A*_ + *R*_*A*_ + *h*_*A*_*P*_*pub*_ is true, and the SECCESPP scheme is bounded.

## Security analysis

In this section, first, we demonstrate the security of the SECCESPP scheme under the random oracle model. Then, we use the formal analysis tool ProVerif to formally analyze privacy. Finally, we provide proof of the unforgeability of the SECCESPP scheme.

### Security under the random oracle model

The SECCESPP scheme is demonstrably secure under the random oracle model in [35] and can resist adaptive chosen message attack. The possible attacks are divided into two types.

TYPE 1: The attacker does not have access to the primary key. However, the attacker can request or replace the user public key. As discussed above, we impose several natural restrictions on TYPE 1: (1) The attacker cannot extract the private key for *ID*_*i*_ at any point. (2) The attacker cannot request the private key for and identity if the corresponding public has already been replaced.

TYPE 2: The attacker does have access to privacy, but cannot request or replace the user public key. The restrictions on this type of attacker are as follows: (1) The attacker cannot replace public keys at any point. (2) The attacker cannot extract the private key for *ID*_*i*_ at any point.

The proofs for the two types of attacks are similar. Hence, we only present the proof for the attacker who does not access the primary key but can request or replace the user public key.

**Definition (ECDLP)**: For a random number $x\in Z_{n}^{*}$, given two elements *P*, *Q* such that *Q* = *x* * *P*, the goal of the ECDLP is to calculate *x*.

**Theorem**: In the random oracle model, the SECCESPP scheme is secure if the ECDLP is intractable.

**Proof**: Assume that attacker B who attacks the SECCESPP scheme. Let attacker B construct algorithm F to solve the ECDLP problem.

**Initialization Phase**: F initializes *P* and *Q* and transmits the public parameters to attacker B. The public parameter is *params* = {*F*_*p*_, *E* / *F*_*p*_, *G*, *P*, *P*_*pub*_ = *Q*, *H*_1_, *H*_2_, *H*_3_, *H*_4_}.

**Queries Phase**: Attacker B executes the following queries, and F adaptively responds to these queries.

1. **User** query: When attacker B performs a user query on *ID*_*i*_, challenger F selects a random number *t* ∈ {1, 2, …, *q*_*c*_}. Then, the pseudo-code is executed.
  **User query** pseudo-code
  1:  if (*i* ≠ *t*) {
  2:   F selects $a,b,c\in Z_{n}^{*}$, *s*_*i*_ · *P* = *R*_*i*_ + *h*_*i*_ · *P*_*pub*_;
  3:   Computes *R*_*i*_ = *a* · *P*_*pub*_ + *b* · *P*, *P*_*i*_ = *c* · *P*, *s*_*i*_ = *b*, *x*_*i*_ = *c*, *h*_*i*_ = *H*_1_(*ID*_*i*_, *R*_*i*_, *P*_*i*_) ← −*a* mod *n*;
  4:    Returns (*ID*_*i*_, *R*_*i*_, *P*_*i*_, *s*_*i*_, *x*_*i*_, *h*_*i*_)and adds into $L_{H_{1}}$;}
  5:   else{
  6:   F selects $a,b,c\in Z_{n}^{*}$;
  7:    Computes *R*_*i*_ = *a* · *P*_*pub*_ + *b* · *P*, *P*_*i*_ = *c* · *P*, *s*_*i*_ = *x*_*i*_ = ⊥, *h*_*i*_ = *H*_1_(*ID*_*i*_, *R*_*i*_, *P*_*i*_) ← −*a* mod *n*;
  8:   Adds (*ID*_*i*_, *R*_*i*_, *P*_*i*_, *s*_*i*_, *x*_*i*_, *h*_*i*_) to *C* − *List*
  9:   Adds (*ID*_*i*_, *R*_*i*_, *P*_*i*_, *h*_*i*_) to $L_{H_{1}}$;
  10:   Returns (*ID*_*i*_, *R*_*i*_, *P*_*i*_, *h*_*i*_) to attacker B;}
2. **Partial Key Extraction** query: If *ID*_*i*_ does not exist, ⊥is output. If *ID*_*i*_ exists and (*i* ≠ *t*), some of the private keys are returned. Otherwise, F stops.
3. **Public Key Replacement** query: If *ID*_*i*_ does not exist, ⊥is output. Otherwise, B replaces user *ID*_*i*_’s public key *PK*_*i*_ with $PK_{i}^{\prime }$ and adds it into *L*_*R*_.
4. **Secret Value** query: When B executes, it is inputs *ID*_*i*_. If *ID*_*i*_ does not exist, ⊥ is output. If *ID*_*i*_ exists and (*i* ≠ *t*), *x*_*i*_ = *c* is sent to B. If (*i* ≠ *t*), then the process stops and returns ‘failure’.
5. **Digest Calculation** query: B executes the summary computation query on *ω*_*i*_ = (*v*_0_, *R*, *PK*_*i*_). If *ID*_*i*_ does not exists, ⊥is output. Otherwise, for the i-th *H*_3_ query, if *ID*_*i*_ ≠ *ID*_*t*_, F queries whether (*ID*_*i*_, *R*_*i*_, *P*_*i*_, *s*_*i*_, *x*_*i*_, *h*_*i*_) exists in *C* − *List*; If it exists, F picks $h\in Z_{n}^{*}$ randomly, and *h* = *H*_4_(*v*_0_, *R*, *PK*_*i*_), whereas if it does not exists, the user’s public key has been replaced. In this case, F generates $a,b,c\in Z_{n}^{*}$, and lets *s* = *a*, *R* = *a*^−1^*h*_*i*_*P*_*pub*_ and *h* = *H*_4_(*v*_0_, *R*, *PK*_*i*_) ← *a*^−1^(*r*_*i*_ + *x*_*i*_). If *ID*_*i*_ = *ID*_*t*_, F picks *ξ* ∈ {0, 1} at random and sets Pr[*ξ* = 1] = *ξ* and Pr[*ξ* = 0] = 1 − *ξ*. If *ξ* = 0, F generates $a,b,c\in Z_{n}^{*}$ and *h* = *H*_4_(*v*_0_, *R*, *PK*_*i*_) ← *a*^−1^(*r*_*i*_ + *x*_*i*_). If *ξ* = 1, F generates $a,b,c\in Z_{n}^{*}$ and lets *h* = *H*_4_(*v*_0_, *R*, *PK*_*i*_). Finally, F returns *h* to B and adds (*ID*_*i*_, *v*_0_, *R*_*i*_, *PK*_*i*_, *h*) to *C* − *List*.
6. **Signcryption Extraction** query: When B extracts the signcryption of (*v*_0_, *ID*_*i*_, *H*_4_(*ω*_*i*_)), if *ID*_*i*_ does not exist, F outputs ⊥. When *ID*_*i*_ ≠ *ID*_*t*_, if (*ID*_*i*_, *R*_*i*_, *P*_*i*_, *s*_*i*_, *x*_*i*_, *h*_*i*_) exist in *C* − *List*, F signs it with the corresponding private key. However, if (*ID*_*i*_, *R*_*i*_, *P*_*i*_, *s*_*i*_, *x*_*i*_, *h*_*i*_)does not exist in *C* − *List*, that means the user’s public key has been replaced. Thus, F lets *s* = *a*, *R* = *a*^−1^*h*_*i*_*P*_*pub*_ and outputs (*R*, *s*) as the signcryption. When *ID*_*i*_ ≠ *ID*_*t*_, if *ξ* = 1, F returns ‘failure’; otherwise, *ξ* = 0, F lets *s* = *a*, *R* = *a*^−1^*h*_*i*_*P*_*pub*_, and it outputs (*R*, *s*) as the signcryption.

**Forgery**: B stops the queries and outputs a valid signcryption (*R*, *s*^(1)^). If *ID*_*i*_ ≠ ID*, ‘failure’ is declared. Otherwise, the attack is successful. Then, F makes full use of the generalized Forking lemma [36] of certificateless signatures, inputs two different *H*_2_ values, repeats the above process, and obtains two different signatures (*R*, *s*^(2)^) and (*R*, *s*^(3)^). Then, *s*^(*k*)^(*R* + *h*^(*k*)^*P*) = *P*_*i*_ + *R*_*i*_ + *h*_*i*_*P*_*pub*_, *k* = 1, 2, 3. Additionally *R* = *lP*, *P*_*i*_ = *r*_*i*_*P*, *P*_*pub*_ = *xP*; thus, *s*^(*k*)^(*l* + *h*^(*k*)^) = *x*_*i*_ + *r*_*i*_ + *h*_*i*_*x*, *k* = 1, 2, 3.

In the above interrogation process, i, *r*_*i*_, *x* are unknown, but F can solve the three unknowns and output *x*; therefore, the elliptic curve discrete logarithm problem can be solved.

To solve a given instance of the ECDLP, F is required to successfully execute the following events:

T_1_: F does not stop the whole time.

T_2_: (*R*, *s*) is a valid signcryption forgery of $\left(ID^{*},PK_{ID}^{*},m^{*}\right)$.

T_3_: *q*_*s*_ is finite and *ξ* = 1.

The probability of the attack of F being successful is $Adv_{ECDLP}^{F}=\text{Pr}[T_{1}\wedge T_{2}\wedge T_{3}]=\text{Pr}[T_{1}]\cdot \text{Pr}[T_{1}|T_{2}]\cdot \text{Pr}[T_{3}|T_{1}\wedge T_{2}]$.

**Claim1**: If *T*_1_ occurs, during the attack of F, the probability of success of the Partial Key Extraction query is ${\left(1-(1/qc)\right)}^{q_{PKE_{x}}}$, where $q_{PKE_{x}}$ is the number of times some keys are queried.

**Claim2**: The probability of success for the Secret Value query is ${\left(1-(1/qc)\right)}^{q_{VE_{x}}}$, where $q_{VE_{x}}$ is the number of times the secret value is queried.

**Claim3**: The probability of success for the Signcryption Extraction query is $(1-(1/qc)\xi )\geq {\left(1-\xi \right)}^{q_{s}}$, where *q*_*s*_ represents the number of times the signcryption is extracted.

As a result,$\text{Pr}[T1]\geq {\left(1-(1/qc)\right)}^{q_{PKE_{\text{x}}}+q_{VE_{x}}}{\left(1-\xi \right)}^{q_{s}}$. We state that Pr[*T*_2_ | *T*_1_] = *ε*, so Pr[*T*_3_ | *T*_1_ ^ *T*_2_] = *ξ* / *q*_*c*._ We can obtain $Adv_{F}^{ECDLP}\geq {\left(1-(1/q_{c})\right)}^{q_{PKE_{x}}+q_{VE_{x}}}{\left(1-\xi \right)}^{q_{s}}\cdot (\xi /q_{c})\cdot \varepsilon$. At *ξ* = 1 /(*q*_*s*_ + 1), ${\left(1-\xi \right)}^{q_{S}}\cdot \xi$ reaches its maximum value, so $Adv_{F}^{ECDLP}\geq {\left(1-(1/q_{c})\right)}^{q_{PKE_{x}}+q_{VE_{x}}}(1-1/{\left(q_{s}+1\right)}^{q_{s}})(1/q_{c}(q_{s}+1))\varepsilon$. Of course, ${\left(1-(1/q_{c})\right)}^{q_{PKE_{x}}+q_{VE_{x}}}(1-1/{\left(q_{s}+1\right)}^{q_{s}})(1/q_{c}(q_{s}+1))\varepsilon$ is a constant and you cannot ignore *ε*, so you cannot ignore $Adv_{F}^{ECDLP}$, which contradicts the hypothesis. The SECCESPP scheme is secure under the random oracle model in [29].

### Privacy

We analyze the privacy of a signed message in the SECCESPP scheme using the formal analysis tool ProVerif [37,38]. Privacy is modeled as confidentiality. First, Applied PI is used to formalize the SECCESPP scheme. Then, ProVerif is used for analyzing privacy.

#### Function and equational theory

**Fig 2:**
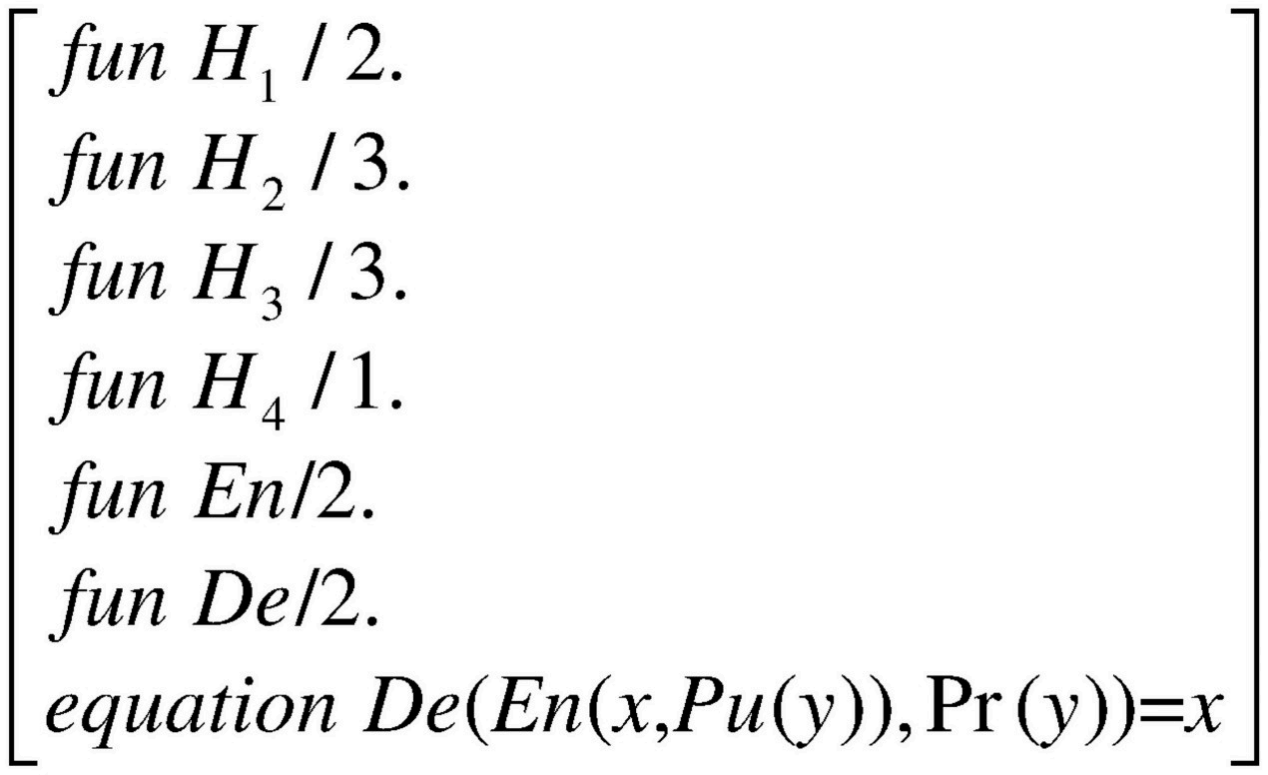
Function and equational theory.

The functions and equations used in the modeling process are described in this section. We use the Applied PI calculus to formalize the SECCESPP scheme. Fig 2 depicts the function and equational theory.

The function and equational theory of the SECCESPP scheme mainly includes the public key encryption algorithm *En*(*x*, *Pu*) encrypt the message *x* with public key *Pu* and the decryption algorithm *De*(*y*, *Pu*) decrypt the message which the *En*(*x*, *Pu*) encrypted with private key *Pu*. The function *Pu*(*y*) accepts private value *y* as input and produces public key as output. The function Pr(*y*) accepts private value *y* as input and produces private key as output.

### Process

**Fig 3:**
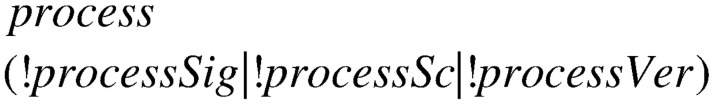
Main process.

**Fig 4:**
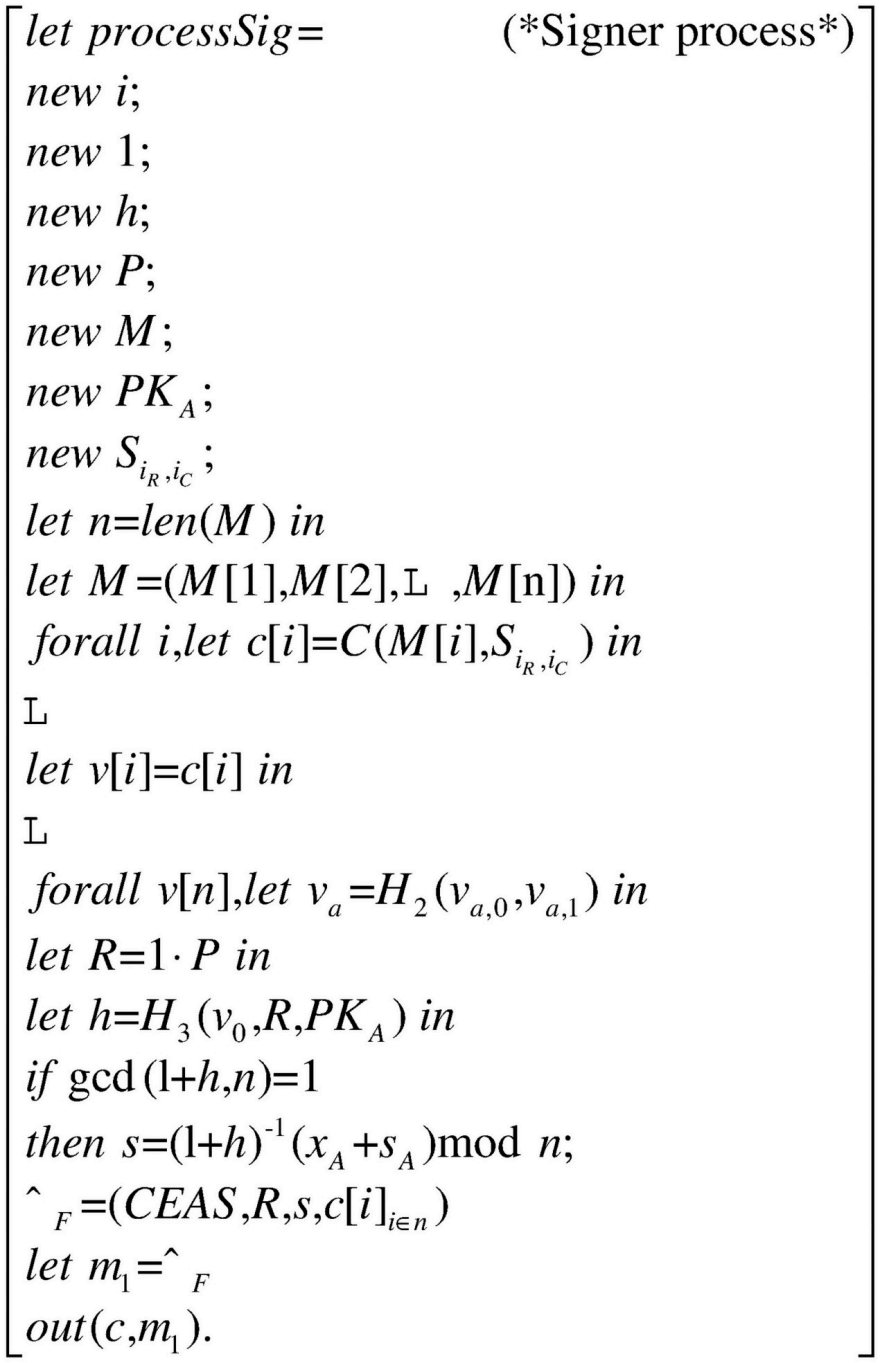
Process processSig.

**Fig 5:**
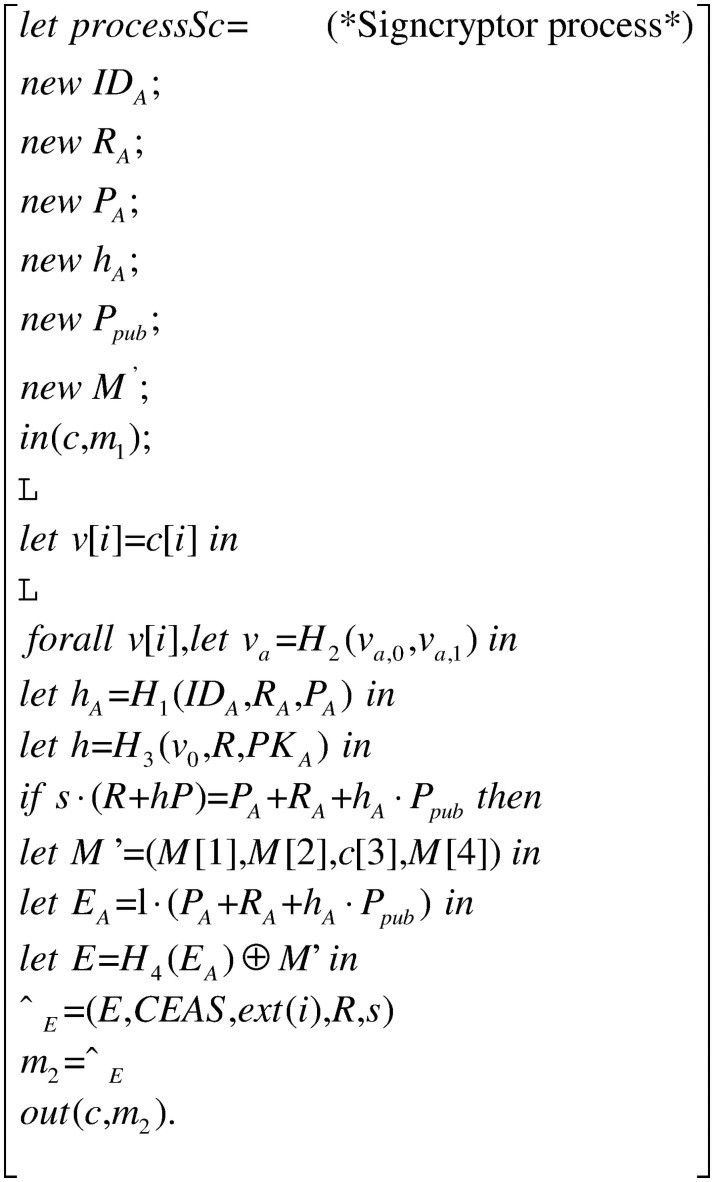
Process processSc.

**Fig 6:**
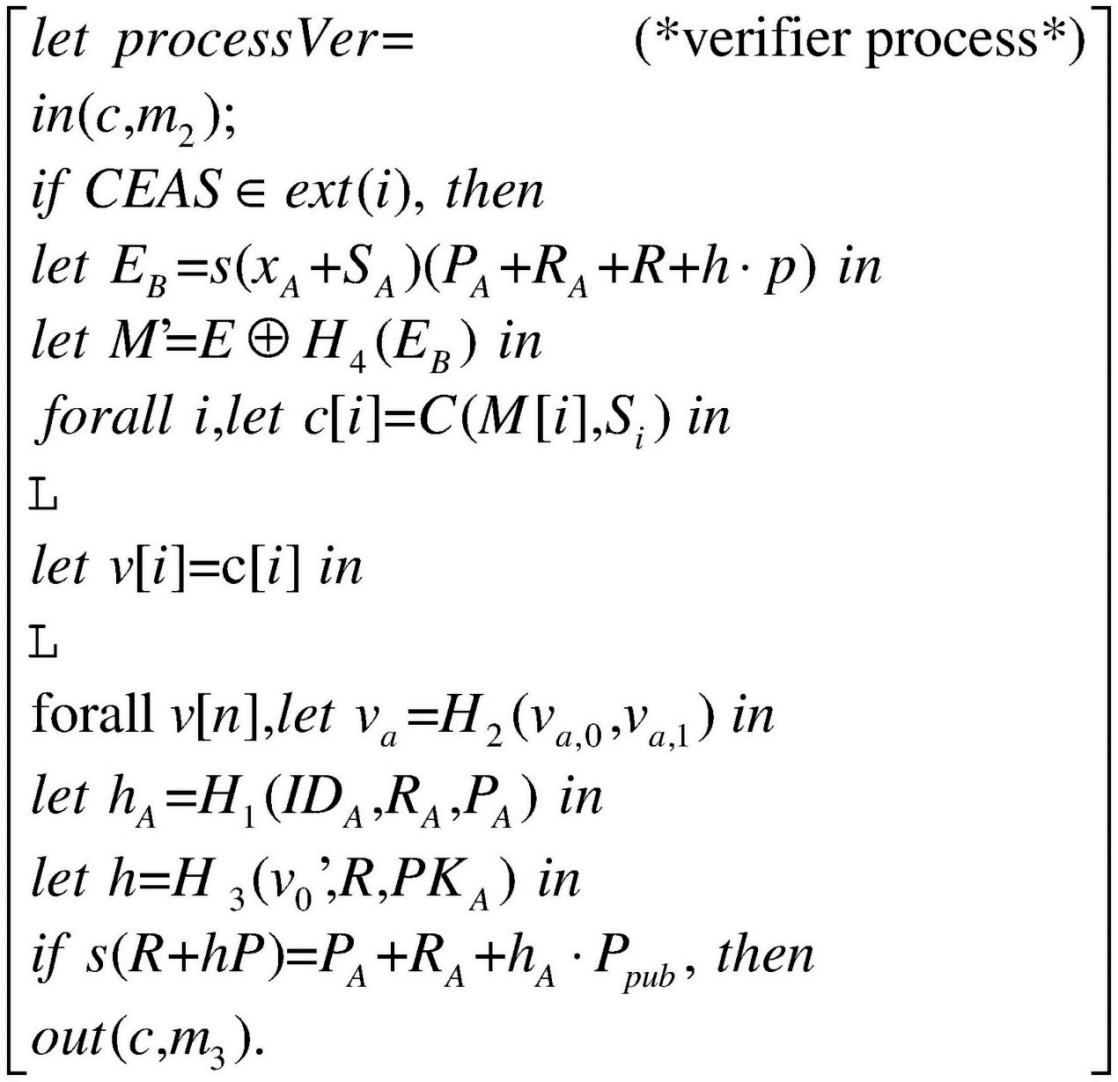
Process processVer.

The whole process in Fig 3 consists of three processes: the signer process *processSig* in Fig 4, the signcryptor process *processSc* in Fig 5 and the verifier process *processVer* in Fig 6. They constitute the main process together, as shown.

The signer first divides the entire message into *n* blocks, and then generates a commitment for each block. After that, the signer calculates *R* and *h*. Then, the signer compares the value gcd() and ‘1’. If they are equal then forwards the signature *σ*_*F*_ to the signcryptor process *processSc* in form of the message *m*_1_ through the public channel *c*.

The signcryptor receives the message *m*_1_ from the signer process through the public channel *c*. Then it extracts the extracted message blocks and the correspondent commitments. After that, the signcryptor calculates *h* and *h*_*A*_. Then it compares the value *s* · (*R* + *hP*)with *P*_*A*_ + *R*_*A*_ + *h*_*A*_ · *P*_*pub*_. If they are equal then encrypts the extracted message *M*′ and follows the content extraction signcryption *σ*_*E*_ to the verifier process *processVer* in form of the message *m*_2_ through the public channel *c*.

The verifier receives message *m*_2_ from the signcryptor process through the public channel *c*. Then it decrypt *M*′ and *v*[*i*], and verifies the signcryption by the equation *s*(*R* + *hP*) = *P*_*A*_ + *R*_*A*_ + *h*_*A*_*P*_*pub*_. Finally, the verifier inputs message *m*_3_ as the verification results.

#### ProVerif for automatic privacy verification

**Fig 7:**
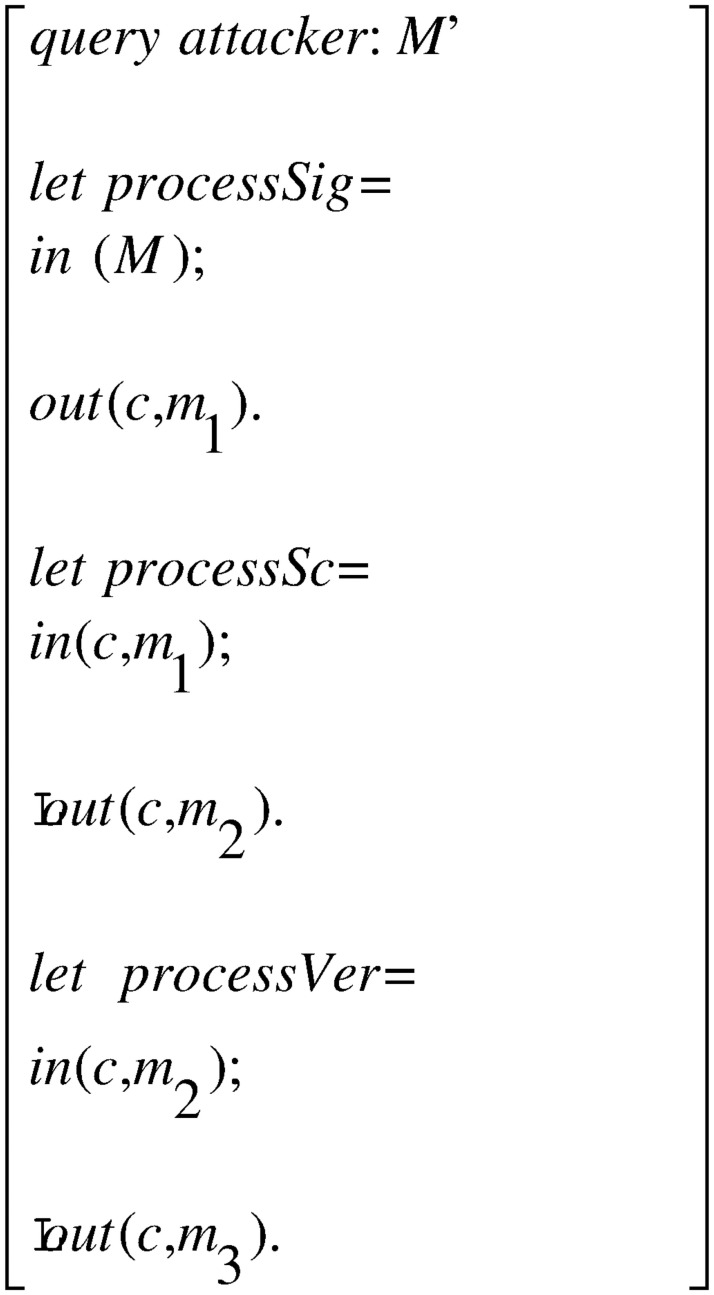
Model for event query.

The privacy of the SECCESPP scheme is modelled as the confidentiality of the signed message *M*. query attacker: *M*′ is used to model the confidentiality of the signed message. and is added into formal model in Fig 7.

#### Result analysis

**Fig 8:**
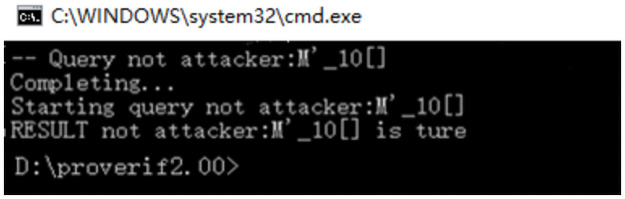
The result of the privacy analysis.

The result in Fig 8 is true, and the SECCESPP scheme has privacy because *M*′ is encrypted before it is sent to the verifier. Attackers can only get encrypted message block, thus, privacy is guaranteed.

### Unforgeability

In a certificateless public key cryptosystem, KGC generates a partial private key, and the user generates a secret value and generates a full private key and the public key respectively according to the secret value and the partial private key. This method solves the key management issue in identity-based cryptosystem and the certificate problem in PKI cryptosystem. It is possible for KGC to forge the user’s signature because KGC holds part of the user’s private key. Therefore, we divide the attack model of the SECCESPP scheme into two types:

Adversary *A*_*I*_: ordinary user attack. In this attack, the attacker cannot obtain the master key but can replace the public key.

Adversary *A*_*II*_: malicious KGC attack. In this attack, the attacker has the master key and can generate any part of the user’s private key, but it is specified that the user’s public key cannot be replaced.

Since Adversary *A*_*I*_ type is similar to Adversary *A*_*II*_ type but Adversary *A*_*II*_ type is more representative, we provide a proof of unforgeability for the Adversary *A*_*II*_ type.

**Theorem**: If Adversary *A*_*II*_ can output a valid content extraction signcryption *σ*_*E*_ and has not performed a Signcryption Extraction query, then the attacker succeeds, that is, the SECCESPP scheme is broken.

**Lemma**: If Adversary *A*_*II*_ wins the game by at least the probability of *ε* after *q*_*k*_ User query, *q*_*PK*_ Key Extract query and *q*_*s*_ Signcryption Extraction query within a bounded time, then the SECCESPP scheme is said to be unforgeability under an adaptive chosen message attack.

**Game**: The SECCESPP scheme in the Adversary *A*_*II*_ case of the adaptive chosen message attack game, which is between challenger C and Adversary *A*_*II*_.

**Proof**: The security model of unforgeability consist of three phases: Setup Phase, Queries Phase and Forgery Phase. In Queries phase, adversary *A*_*II*_ performs multiple queries including User query, Key Extraction query and Signcryption query. challenger C gives corresponding responses.

**Setup Phase**: Adversary *A*_*II*_ makes multiple queries, challenger C maintains lists *l*_1_ − *l*_3_ that are empty initially.

Initialization: Challenger C runs the Initialization algorithm. Input security parameter *k*, challenger C generates $x\in Z_{n}^{*}$, system master key *P*_*pub*_ and the system parameter *params*, and sends them to Adversary *A*_*II*_. The *params* = {*F*_*p*_, *E* / *F*_*p*_, *G*, *P*, *P*_*pub*_ = *Q*, *H*_1_, *H*_2_, *H*_3_, *H*_4_}. Set $a\in Z_{n}^{*}$.

**Queries Phase**: Adversary *A*_*II*_ executes the following queries, and challenger C adaptively responds to these queries.

1. **User** query: When Adversary *A*_*II*_ presents query on *ID*_*t*_, challenger C maintains a hash list *H*_1_ − *list* that is initially empty including two-tuples (*ID*_*t*_, *Q*_*t*_). Challenger C checks whether record exists in a hash list *H*_1_ − *list*. If so, challenger C returns corresponding record, else challenger C makes the following responses:
$$Q_{t}=\left\{ \begin{array}{l} (ID_{i},R_{i},P_{i},h_{i}),i=t\\ (ID_{i},R_{i},P_{i},s_{i},x_{i},h_{i}),i\neq t \end{array}\right.$$ (7)
2. **Key Extract** query: Adversary *A*_*II*_ performs queries on the certificateless public key cryptosystem, *m*_*i*_ represents the identity of the user. Challenge C calculates *ID*_*i*_’s public key *PK*_*i*_ and sends it to Adversary *A*_*II*_.
3. **Signcryption Extraction** query: Adversary inputs *ID*_*i*_ and a new message *M*′, and challenger C runs Signcrpytion-Extraction algorithm according to private key *SK*_*i*_ and sends result to the Adversary *A*_*II*_.

**Forgery Phase**: Adversary *A*_*II*_ inputs (ID*, PK*, *M*′, σ*). To win this game, Adversary *A*_*II*_ is required to successfully execute the following events:

T_1_:$\sigma_{E}^{*}$ is a valid signcryption forgery of $\left(ID^{*},PK_{ID}^{*},M\prime \right)$.

T_2_: ID*never executed Key Extract query.

T_3_: $\left(ID^{*},PK_{ID}^{*},M\prime \right)$ no executed Signcryption Extraction query.

Let *Succ*_*UF−CMA*_ = *ε* = Pr[*T*_1_ ^ *T*_2_ ^ *T*_3_] be the probability that Adversary *A*_*II*_ wins the game.

**Claim1**: The probability of success for the Signcryption Extraction query is $(1-{\left(1/qc\right)}^{q_{s}}\geq {\left(1-\xi \right)}^{q_{s}}$, where *q*_*s*_ represents the number of times the signcryption is extracted and *ξ* ∈ {0, 1}.

**Claim2**: The probability of success for the User query is ${\left(1-(1/qc)\right)}^{q_{k}}$, where *q*_*k*_ is the number of times the private key is queried.

**Claim3**: During the attack of Adversary *A*_*II*_, the probability of success for the Key Extract query is ${\left(1-(1/qc)\right)}^{q_{PK}}$, where *q*_*PK*_ is the number of times public key are queried.

As a result, $\text{Pr}[T1]\geq {\left(1-(1/qc)\right)}^{q_{PK}+q_{k}}{\left(1-\xi \right)}^{q_{s}}$. We say that Pr[*T*_2_ | *T*_1_] = *ε*, so Pr[*T*_3_ | *T*_1_ ^ *T*_2_] = *ξ* / *q*_*c*._ We can obtain $Succ_{UF-CMA}\geq {\left(1-(1/q_{c})\right)}^{q_{PK}+q_{k}}{\left(1-\xi \right)}^{q_{s}}\cdot (\xi /q_{c})\cdot \varepsilon$. At *ξ* = 1 / (*q*_*s*_ + 1), ${\left(1-\xi \right)}^{q_{S}}\cdot \xi$ reaches its maximum value, so $Succ_{UF-CMA}\geq {\left(1-(1/q_{c})\right)}^{q_{PK}+q_{k}}(1-1/{\left(q_{s}+1\right)}^{q_{s}})(1/q_{c}(q_{s}+1))\varepsilon$. Of course, ${\left(1-(1/q_{c})\right)}^{q_{PK}+q_{k}}(1-1/{\left(q_{s}+1\right)}^{q_{s}})(1/q_{c}(q_{s}+1))\varepsilon$ is a constant and you cannot ignore *ε*, so you cannot ignore *Succ*_*UF−CMA*_ (*ε*), which satisfies what Lemma says. Therefore, the SECCESPP scheme has unforgeability.

## Comparison and discussion

In the SECCESPP scheme, we use scalar multiplication on elliptic curves, thereby reducing the number of calculations in the signing and verification process. For efficiency, we compare two aspects of the proposed scheme to those of related schemes in [14–19]. One is the theory calculation aspect. The other is the practical running time aspect. For the theory calculation aspect, the SECCESPP scheme is compared in terms of the following four factors in Tale 1: exponential operation (exp), scalar multiplication (sca), bilinear pairing operation (par) and hash function (has), where *n* is the number of submessages, *m* is the number of the extracted messages, *m*_*CEAS*_ is the number of submessages in the content extraction access structure *CEAS*. The SECCESPP scheme has “(2n+2)has+4sca” and “(m+2)has+2sca” calculation, which are the lowest amounts. Hence, the SECCESPP scheme is highly efficient from the theoretical calculation aspect.

For the practical running time aspect, the hardware platform consists of Intel Core m3-6Y30 @0.90 GHz processor and 8GB memory. The software environment includes Windows 10 operating system for 64 bits and Miracl library [39] for which the parameters are specified as follows: the supersingular elliptic curve *E* / *F*_*p*_: *y*^2^ = *x*^3^ + *x* is selected, in which the embedding degree is 2 and the prime number *p* satisfies 2^510^ < *p* < 2^511^, *p* + 1 = 12*qr*, *q* = 2^159^ + 2^17^ + 1. The Tate pair operation is defined in *E* / *F*_*p*_: *y*^2^ = *x*^3^ + *x*. The scalar multiplication operations on the elliptic curves satisfy *p* = 2^160^ − 2^31^ − 1.

To avoid randomness, the simulation experiments are performed five times to obtain the averaged results. The computing time of the four factors is shown in Table 2. According to Tables 1 and 2, the running time in Table 3 are calculated.

**Table 1:** The number of operation.

scheme	Signcryption and extraction	Verification
**[14]scheme**	(2n+3)has+2exp+1par+4sca	(m+3)has+1par+2sca
**[15]scheme**	(2n+3)has+7exp	(m+3)has+5exp
**[16]scheme**	(2n+3)has+5exp	(m+3)has+3exp
**[17]scheme**	(2n+3)has+4sca	(m+3)has+3sca
**[18]scheme**	(5n)sca	(3m)par
**[19]scheme**	(2n+2)exp+1par	(m+3)par+2exp
**our scheme**	(2n+2)has+4sca	(m+2)has+2sca

**Table 2:** Computing time on operations.

Operations	has	exp	par	sca
**running time**	2.01ms	3.31ms	10.02ms	1.51ms

**Table 3:** Running time.

scheme	Signcryption and extraction/ms	Verification/ms
**[14] scheme**	4.02n+28.71	2.01m+19.07
**[15] scheme**	4.02n+29.20	2.01m+22.58
**[16] scheme**	4.02n+22.58	2.01m+15.96
**[17] scheme**	4.02n+12.07	2.01m+10.56
**[18] scheme**	7.55n	30.06m
**[19] scheme**	6.62n+16.64	10.02m+36.68
**our scheme**	4.02n+10.06	2.01m+7.04

**Fig 9:**
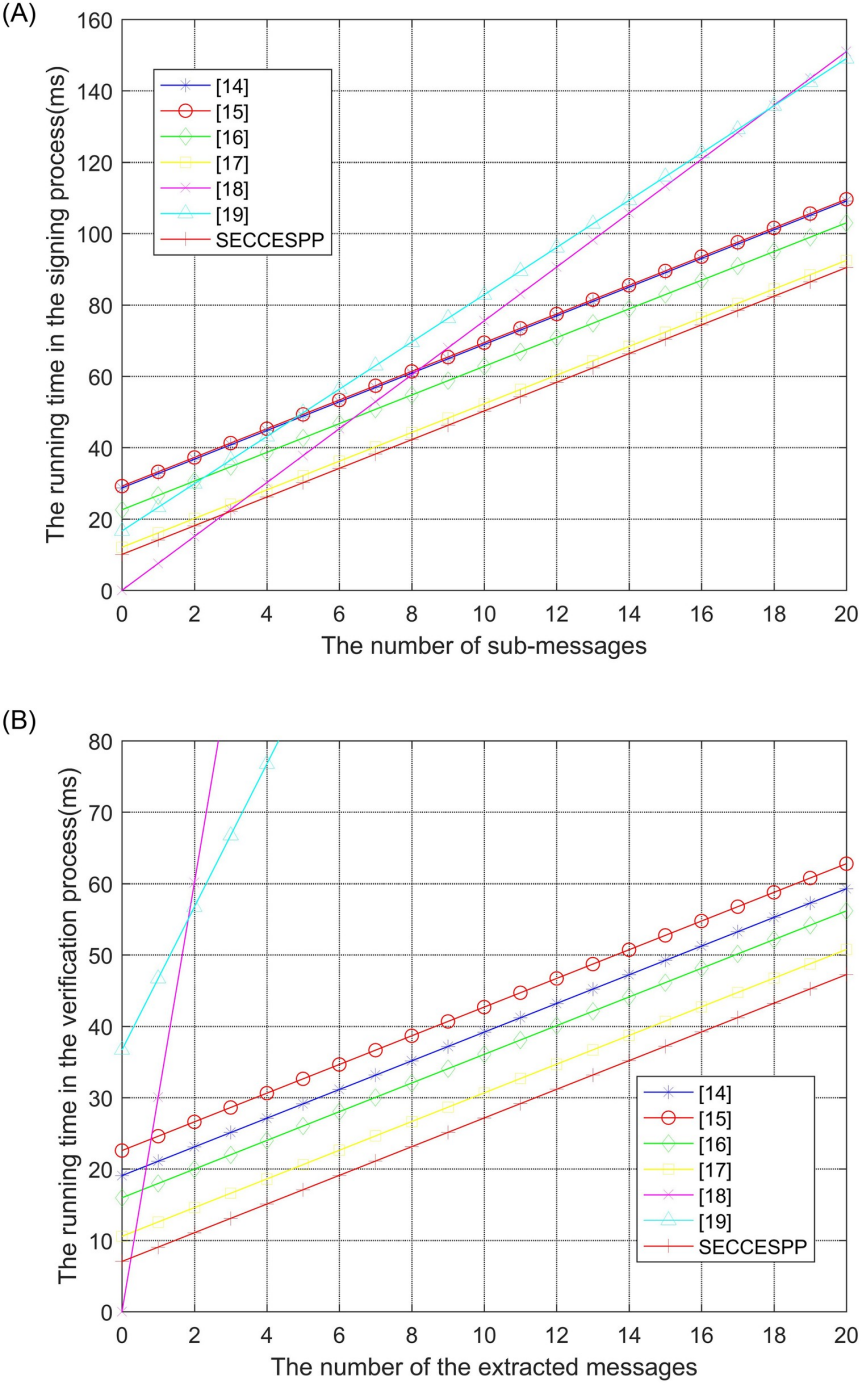
Comparison of running time in the signing and verifying process.

The running time of the scheme depends on the signcryption and extraction time and verification time. From Table 3 and Fig 9, we can clearly see that the scalar multiplication operation on the elliptic curves takes much less time than bilinear pair operation and exponential operation. Hence the analysis result of theory calculation is consistent to analysis result of the practical running time. Therefore, the SECCESPP scheme has higher efficiency than compared scheme.

## Conclusion

To improve the efficiency of and provide privacy protection for content extraction signatures, we proposed the SECCESPP scheme in which the scalar multiplication on elliptic curves is used to replace inefficient bilinear pairing in a certificateless public key cryptosystem, and the signcryption idea is borrowed to provide privacy protection. The SECCESPP scheme is provably secure based on the elliptic curve discrete logarithm problem in the random oracle model. It not only has correctness and privacy, but is also more efficient than related schemes [14–19].

In the future, we will use the SECCESPP scheme to the off-chain data access in blockchain to implement security and privacy.
